# Effects and mechanism of duodenal-jejunal bypass and sleeve gastrectomy on GLUT2 and glucokinase in diabetic Goto–Kakizaki rats

**DOI:** 10.1186/2047-783X-17-15

**Published:** 2012-06-11

**Authors:** Zhou Donglei, Lu Liesheng, Jiang Xun, Zhen Chenzhu, Ding Weixing

**Affiliations:** 1 Department of Gastrointestinal Surgery, Shanghai Tenth People’s Hospital, Shanghai Tongji University, #301 Yanchang Zhong Rd, Shanghai, 200073, China; 2 Department of General Surgery, Affiliated Hospital of Second Military Medical University, #168 Changhai Rd, Shanghai, 200433, China

**Keywords:** Duodenal-jejunal Bypass, Sleeve Gastrectomy, GLUT2, Glucokinase

## Abstract

**Background:**

The study investigated the effects and mechanism of duodenal-jejunal bypass (DJB) and sleeve gastrectomy (SG) on the expression of liver GLUT2 and glucokinase (GCK) in diabetic rats.

**Methods:**

Animal models of Goto–Kakizaki (GK) rats were established for the investigation of DJB and SG. Results of weight, food intake, fasting plasma glucose level, oral glucose tolerance test and insulin were compared. Liver tissues were harvested 8 weeks postoperatively. Reverse transcription-PCR and western blot were used to detect liver GLUT2 and GCK mRNA and protein expression after operation.

**Results:**

Fasting plasma glucose levels of DJB group and SG group in GK rats were markedly declined at 3 days and l, 2, 4, 6, and 8 weeks postoperatively (*P <*0.01), whereas the levels of the sham-operated group only dropped at 3 days and 1 week postoperatively, and there were no significant differences 2 weeks postoperatively (*P >*0.05). In the liver of GK rats, GLUT2 mRNA level and protein expression after DJB were higher than those in sham-operated group and control group. GLUT2 mRNA level and protein expression after SG were significantly lower than those in control group (*P <*0.01). GCK mRNA and protein experienced similar expression change.

**Conclusions:**

Both DJB and SG can decrease the plasma glucose levels of GK rats, whereas they have different effects on the expression of liver GLUT2 and GCK.

## Background

According to WHO data, there were 175 million diabetic patients in 2000, of which 90% were type 2 diabetes, and the world incidence of diabetes showed a significant upward trend year by year that is expected to double by the year 2025 [1,2]. Complications in cardiovascular, brain, kidney and other vital organs caused by diabetes threaten patients’ health and life directly [3]. Although drugs can control blood glucose level, they rarely make patients’ blood glucose level become normal and do not prevent diabetes complications. Strict diet and repeated fluctuations in blood glucose levels place a continuous mental stress on patients and affects their quality of life.

Gastrointestinal reconstruction surgery is currently receiving much attention in type 2 diabetes treatment. Investigators had found that many type 2 diabetes patients achieved clinical resolution after bariatric surgery of morbid obesity, which suggested that the diabetes may be cured by surgery. It was found that 95% diabetes patients with normal body mass index have controlled blood glucose after surgery [4]. Many studies suggest that hormonal effect resulting from the anatomical changes in the gastrointestine is the main mechanism of long-term stability of controlling blood glucose. However, there are studies that consider the high metabolic status and low calorie intake to be the major factors in controlling plasma glucose [5].

At present, the mechanism research of surgical treatment of diabetes mainly focuses on a variety of hormonal changes produced by the surgery, including Grelin, Obstatin, PYY, and GLP-1 [6,7], but studies on the absorption, decomposition and use of glucose after surgery are rare. In the past, many studies increased the expression of glucose transport protein through genetically modified methods and obviously improved blood glucose levels. However, there are no reports on the expression level changes of glucose transporter protein through gastrointestinal surgery.

In this experiment we use non-obese diabetic Goto–Kakizaki (GK) rats as the experimental animal. There are two methods: duodenal-jejunal bypass (DJB) reduces absorption, and sleeve gastrectomy (SG) limits intake. We aim to explore the effects of the two surgical methods on liver glucose transport protein 2 and liver glucokinase (GCK) expression to understand the role of surgery in glucose metabolism, and to study the mechanism of surgical treatment of diabetes.

## Methods

### Animals

The GK rat is a non-obese Wistar substrain which develops type 2 diabetes mellitus (DM) early in life. The model was developed by Goto and Kakizaki at Tohoku University, Japan in 1975. The GK rat is produced by selective breeding (with glucose intolerance as a selection index) repeated over many generations, starting from a non-diabetic Wistar rat colony [8]. GK rats are widely used in non-insulin-dependent DM (i.e. type 2 diabetes).

Male 8-week-old GK rats were purchased from Shanghai Laboratory Animal Center (Shanghai, China). Rats were housed individually in a controlled environment and maintained on a 12:12 artificial light/dark cycle with free access to tap water and a 5.5% fat rat chow diet (Shanghai Laboratory Animal Center, Shanghai, China) throughout the experiment. The energy density of the forage is 4.6 Kcal/Kg. All studies were approved by the Shanghai Laboratory Animal Care Committee and carried out in compliance with National Institute of Health standards.

### Experimental design

Thirty two GK rats randomly underwent one of the following procedures(8 for each condition): duodenal jejuna bypass group (DJB, *n =* 8), SG group (SG, *n =* 8), sham-operated group (SHAM, *n =* 8) and control group (CONT, *n =* 8). No significant differences were found among the GK rats before surgery in terms of weight, food-intake, fasting plasma glucose concentration and mean plasma glucose.

After the rats had been acclimatized for 1 week, we measured food intake daily and body weight twice weekly. The preoperative baseline oral glucose tolerance test (OGTT) was performed 1 week before the surgical treatment and at Week 4 and Week 8 after the surgical treatment. At 10 weeks old, the GK rats were randomly assigned to one of the following procedures: DJB, SG, sham operation, and no operation, respectively. Fasting plasma glucose levels were determined on tail vein blood in conscious rats at 3 days and 1, 2, 4, 6, and 8 weeks postsurgery. Plasma levels of insulin were measured at 2, 4, 6, and 8 weeks postoperatively. All the rats were sacrificed to draw tissues for additional experimenting when the rats were 32 weeks old.

Under light ether anesthesia, blood samples were drawn from the retro-orbital venous plexus using heparinized capillary tubes. After centrifugation, plasma samples were stored at −80 °C for later determination of plasma insulin levels.

Liver tissues were separated into small pieces of 1 g weight after having been fully washed with PBS. The tissues for RNA detection were immersed into frozen pipe with RNA stabilization solution RNALater (Qiagen, Valencia, CA, USA), and tissues for protein detection were immersed into frozen pipe with protease inhibitors. The frozen pipes were immediately put into liquid nitrogen and stored at −80 °C.

### Surgical procedures

After adaptive feeding of 2 weeks without any intervention, all GK rats were randomly divided into four groups. Rats undergoing any operation were made to fast but were given free access to tap water overnight. At half an hour before surgery, 50 mg/kg ceftriaxone was injected into muscle and inhalation anesthesia with 0.5% pentobarbital sodium in enterocoelia.

DJB surgery procedure: first, we degermed the skin, made a 2.5 cm midline abdominal incision, identified the structures, and separated the vessels from the pylorus. Then, we transected the pylorus, and sutured continuously with 5–0 silk to close the duodenum stump. We transected the jejunum at a distance of 10 cm from the ligament of treitz, and connected the distal jejunum to the pylorus end-to-end anastomosis with 7–0 absorbable silk, an end-to-side anastomosis with the proximal limb and jejunum 15 cm distally was made with the same silk. The gastric volume was preserved during the procedure.

SG surgery: first, the same incision as was done in the DJB surgery was made, and gastric omentum was dissociated to disclose gastric cardium. To dissect the fundus and greater curve, ligation with 6–0 silk of the short vessels towards the spleen and of the gastroepiploic vessels in the region of the antrum was needed. Then, the vessels of the greater curvature were cauterized with a thermocautery, from the cardia all the way to the pylorus. This arrangement defined the line of incision for the longitudinal SG. This line covered the entire lumen and much of the gastric fundus in which 70% of total stomach was removed. After exeresis, the peritoneal cavity was cleaned with saline before the gastrorrhaphy was conducted with an invaginating continuous polypropylene (Prolene 6–0; Ethicon, Somerville, NJ, USA) hand-sewn suture (Schimieden pattern) from the fundus to the antrum. Hemostasis and suture-line integrity were checked, and an additional stitch was applied when necessary.

Sham surgeries involved the same incisions, transections and reanastomosis of the gastrointestine at multiple sites corresponding to those in SG and DJB. After transection, the intestines were immediately anastomosed. When needed, operative time was prolonged to ensure a degree of anesthesiological stress that was equivalent to that undergone by the SG and DJB rats.

CONT groups did not have an operation.

### RNA extraction and real-time reverse transcription-PCR

Total RNA was extracted from cells using the Trizol reagent (Invitrogen, Carlsbad, CA, USA) following the manufacturer’s directions. Reverse transcription was carried out to convert mRNA into complementary DNA following Invitrogen’s protocol. Quantitation of cDNAs was performed by real-time reverse transcription-PCR with the ABI Prism 7300 Sequence Detection System (Applied Biosystems, Foster City, CA, USA).

### Western blot analysis

For western blot detection of GLUT2 and GCK, liver tissues were lysed in the lysis buffer consisting of 50 mmol/L Trizma base (pH 8; Sigma, St. Louis, MO, USA), 1% Triton X-100, 150 mmol/L NaCl, 20 Ag/mL leupeptin, 10 Ag/mL aprotinin, 1 mmol/L phenylmethylsulfonyl fluoride, and 1 mmol/L sodium vanadate. Protein concentrations were determined by the bicinchoninic acid protein assay (Pierce Chemical Co., Rockford, IL, USA) using bovine serum albumin as standard. Equal protein aliquots were resolved by SDS-PAGE, transferred to polyvinylidene fluoride membranes, immunoblotted with primary antibody, and detected with horseradish peroxidase-conjugated secondary antibody (Bio-Rad Laboratories, Hercules, CA, USA) and enhanced chemiluminescence reagent (Amersham Biosciences, Piscataway, NJ, USA).

### Statistical analysis

All statistical procedures were performed using SPSS version13.0. Data were expressed as mean values ± SD. Differences between groups were analyzed by two-way analysis of covariance in combination with the unpaired or paired Student's *t*-test when appropriate. When data did not follow a normal distribution or failed the equal variance test, the Mann–Whitney *U* test was used to analyze differences between groups. Groups were considered to be significantly different at *P <*0.05.

## Results

### Baseline (preoperative) evaluation

Before surgery, no significant differences were found among the diabetic groups as well as the non-diabetic groups in terms of weight, food intake, fasting plasma glucose concentration, and mean plasma glucose during an OGTT (data not shown).

### Weight

**Figure 1:**
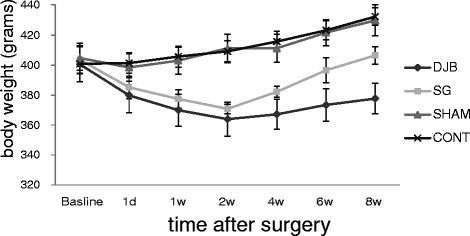
**Mean ± SD body weights of rats.** Both SG and DJB groups show less weight gain compared with SHAM and CONT animals (*P <*0.001). DJB group showed a more significant weight loss than SG group.

The study showed that there were no statistical differences in body weight among groups before surgery(*P >*0.05) (Figure 1). Both SG and DJB surgery lead to significant weight loss compared with the SHAM rats and CONT group rats (*P <*0.001) at the first 2 weeks after surgery. The two groups started regaining body weight approximately the 14th postoperative day. The mean weights of CONT and SHAM groups did not differ from one another at any period, and had a steady growing trend.

### Food intake

**Figure 2:**
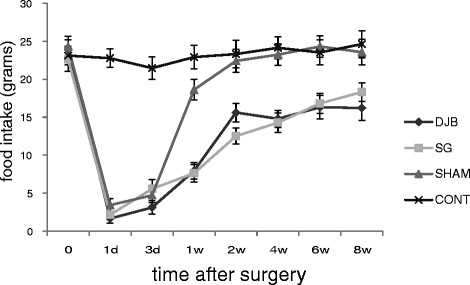
**Mean ± SD food intake of rats.** Both SG and DJB groups show less food intake compared with SHAM and CONT animals.

There were no significant differences among the groups of GK rats on preoperative food intake (Figure 2). At the first day postoperatively, food intake of DJB, SG and SHAM groups had significant decreased (*P <*0.001), whereas the CONT group remained stable. After that, food intake of the three groups returned gradually. At 2 weeks postoperatively, food intake of SHAM group approached that of the CONT group. No significant differences were found between the two groups at Week 8 postoperatively (*P >*0.05). The food intake of DJB and SG groups were significant lower than SHAM and CONT groups.

### Fasting plasma glucose level

**Figure 3:**
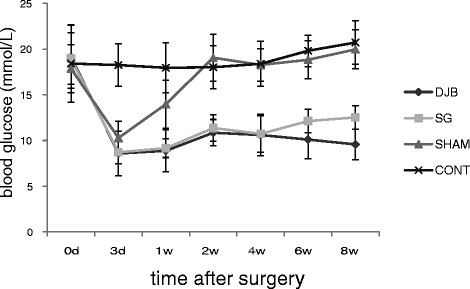
**Mean ± SD fasting glycemia.** Mean fasting glycemia remained constantly lower in SG and DJB rats compared with SHAM and CONT rats (*P <*0.001). There were no significant differences between SHAM rats and CONT rats (*P >*0.05).

Fasting plasma glucose levels of DJB group and SG group in GK rats were markedly declined at 3 days and l, 2, 4, 6, and 8 weeks postoperatively (*P <*0.01), whereas those of the SHAM group only dropped at 3 days and 1 week postoperatively, and there were no significant differences 2 weeks postoperatively (*P >*0.05) (Figure 3).

### OGTT

**Figure 4:**
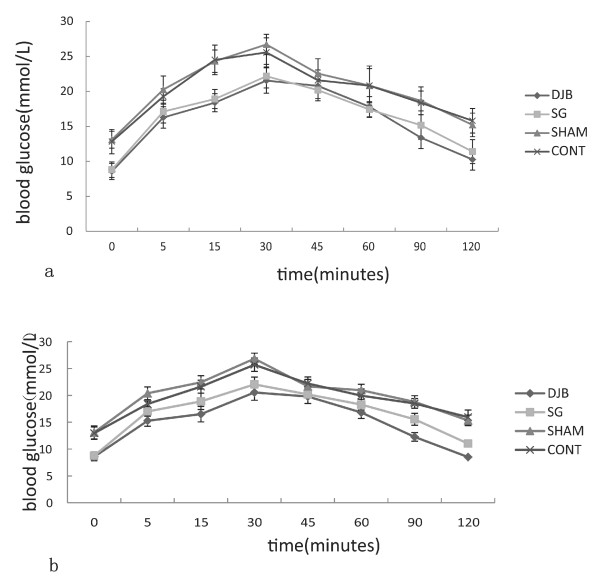
**a. OGTT was performed 4 weeks after operation. b.Another OGTT was taken 8 weeks after operation.** It showed an improvement of glucose tolerance in both SG and DJB groups compared with the SHAM rats at 4 weeks as well as 8 weeks after operation (*P <*0.001). SG group showed a better glucose tolerance than DJB group (*P <*0.001).

This study showed that at Week 4 (Figure 4a) and Week 8 (Figure 4b) after surgery, OGTT were improved in both SG and DJB rats compared with the SHAM and CONT rats (*P <*0.001). The improvement of OGTT was better in SG rats than in DJB rats.

### Insulin level

**Figure 5:**
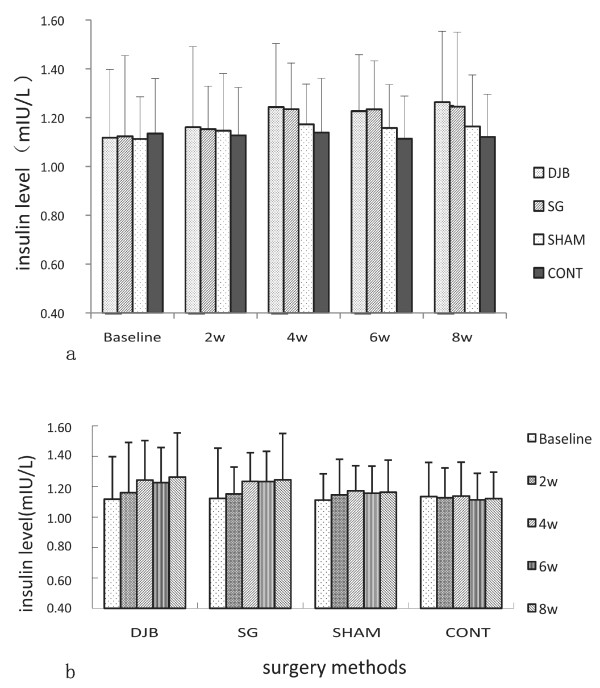
**a. The variation tendency of insulin level at different times in GK rats. b. The variation tendency of insulin level of different surgical treatment in GK rats.** No statistical differences were observed among different times as well as different surgical treatments.

Insulin was measured before and at 2, 4, 6 and 8 weeks after operation. SG and DJB rats displayed inconspicuous improvements in insulin levels after the operations compared with SHAM and CONT rats, but no statistical differences were observed (*P >*0.05) (Figure 5a, Figure 5b).

### Expression level of liver GLUT2

**Figure 6:**
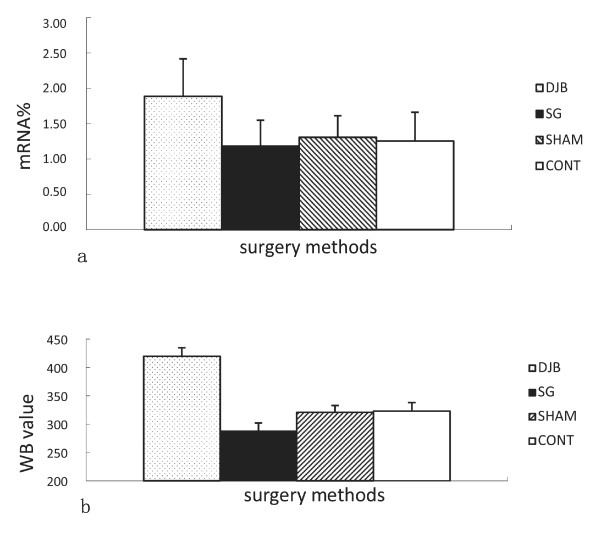
**a. Mean ± SD mRNA expression levels of liver GLUT2. b.Mean ± SD protein expression levels of liver GLUT2.** DJB operation upregulated the expression of liver GLUT2 in GK rats, whereas SG downregulated the expression.

The expression levels of liver GLUT2 mRNA in DJB group were significantly upregulated compared with CONT group (*P <*0.05), however, the GLUT2 mRNA expression levels of SG did not show statistical difference compared with SHAM and CONT groups (*P >*0.05) (Figure 6a). Expression of liver GLUT2 protein in DJB was significantly higher than in CONT (*P <*0.001), whereas SG was significantly lower than in CONT (*P* <0.001), and there was no significant difference between SHAM and CONT (*P <*0.05) (Figure 6b).

### Expression level of liver GCK

**Figure 7:**
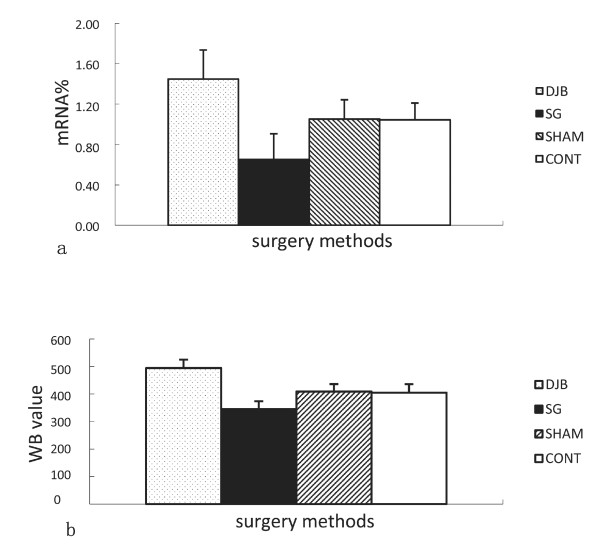
**a. Mean ± SD mRNA expression levels of liver GCK. b.Mean ± SD protein expression levels of liver GCK.** DJB operation upregulated the expression of liver GCK in GK rats, whereas SG downregulated the expression.

The mRNA and protein expression of liver GCK in DJB was significantly higher than in CONT (*P <*0.01), and in SG it was significantly lower than in CONT (*P <*0.001) (Figure 7a, Figure 7b).

## Discussion

In this present study, we performed gastrointestinal surgery on GK rats to investigate the effects and mechanism of DJB and SG on the expression of liver GLUT2 and GCK in diabetic rats. We found that both DJB and SG can decrease the plasma glucose levels of GK rats, whereas there are different effects on the expression of liver GLUT2 and GCK. To our knowledge, this is the first report on the expression level changes of glucose transporter protein through gastrointestinal surgery.

Currently, there is an exponential increase in the prevalence of type 2 diabetes within the population worldwide. Current therapies including diet, exercise, behavior modification, oral hypoglycemic agents, and insulin [9-11] rarely return patients to euglycemia. For this reason, an efficient way to treat patients with diabetes is needed. There is evidence that bariatric surgery is an effective form of therapy for type 2 diabetes. It is reported that SG and DJB for the treatment of type 2 diabetes in obese patients are effective treatments for diabetes [12-14], and they restore normal concentrations of plasma glucose, insulin, and glycosylated hemoglobin in 80% to 100% of patients [15-17]. However, for normal weight or slightly overweight patients with type 2 diabetes, the debate on whether cutting most of the stomach is an effective way to treat diabetes continues.

Recent reports that glycemic control often occurs long before significant weight loss [15,18] suggesting that the control of diabetes may be a direct effect of the operation rather than a secondary outcome of the amelioration of obesity-related abnormalities. From this experiment, the changes of diet and weight of SG and DJB groups were consistent, they declined at first and then rose slowly, but both the food intake and weight of SG and DJB were significantly lower than that of the CONT group. Furthermore, these effects were not seen in the sham-operated animals despite similar operative time, and the same postoperative food intake rates. This suggests that SG and DJB could change the weight of a patient even in a non-obese animal model.

Similar effects with SG and DJB on OGTT, fasting plasma glucose level, and plasma insulin level were observed. Similar to previous observations, these surgeries achieved normal concentrations of fasting glycemia and fasting plasma insulin [13,18-20], restored insulin sensitivity [18,21-23], and prevented progression in impaired glucose tolerance [21,22]. The results of SG and DJB were significantly different from those of the CONT group and the SHAM group. However, SG and DJB rats displayed inconspicuous improvements in insulin levels after the operations compared with SHAM and CONT rats, but no statistical differences were observed. These findings are consistent with previous studies in humans in which the control of plasma glucose and insulin has occurred before substantial weight loss after bariatric surgery [24].

Previous studies [25,26] showed that glucose can increase the level of GLUT2 mRNA. Glucose must pass through the β-cell membrane to the cytoplasm before the GCK can work, and the process is regulated by GLUT2. This transmembrane transport is unlimited in physiological circumstances, but glucose-stimulated insulin secretion is impaired in the DM state, and the expression level of GLUT2 is significantly reduced. GCK makes liver absorb glucose more easily during the high level of glucose. Liver GCK deficiency can affect the function of β-cell and impair insulin secretion. A decrease in the production of GCK or in the activity of GCK can reduce glucose utilization, block glycogen synthesis, and lead to elevated glucose levels [27]. The expression of GLUT2 and GCK are affected by many factors. We used animal experiments, and a unified diet and rearing environment, so that the level of glucose metabolism of the liver can be investigated. The expression of liver glucose transporter protein and liver GCK of the DJB group increased significantly compared with the SHAM group. Though DJB did not alter stomach capacity, gastrointestinal hormones were changed after gastrointestinal anatomy which led to poor appetite, so food intake in that group was low. The food intake in SG was higher than in DJB at 8 weeks after surgery; however, the expression of GCK and GLUT in SG was lower than in DJB.

This study showed that DJB and SG on GK rats improved blood glucose levels. The blood glucose level of the SHAM group was improved in the short term, but it returned to the preoperative level 2 weeks after surgery. DJB and SG improved insulin levels slightly in GK rats, but the differences were not statistically significant. The mRNA and protein expression of liver GCK of the DJB group were significantly increased, however, the expression of GCK of the SG group was significantly decreased. So we can conclude that the mechanism of glucose metabolism in different surgical methods is completely different.

Stomach capacity is still intact after DJB, however, changes in the gastrointestinal anatomy lead to some changes in gastrointestinal hormones: it amplifies the function of insulin, promotes the expression of GCK and enhances glycogen synthesis and thereby the blood glucose is reduced [28]. After SG surgery, due to restricted food intake, the amount of glucose entering the blood was significantly reduced, which led to low levels of glucose metabolism in the liver. Because the gastrointestinal structure was unaffected, the gastrointestine did not produce hormones that activate GCK, and GCK expression was therefore lower than the SHAM group and the CONT group. In this study, blood glucose levels were not consistent with high expression of GCK after DJB surgery in GK rats, which shows that the hormone effect promoted over expression of GCK after gastrointestinal bypass surgery.

## Conclusions

The results suggest that different surgical methods can produce completely different changes in glucose metabolism. For GK rats with a normal body mass index, just limiting food intake does not improve the regulation of blood glucose. Both DJB and SG can decrease the plasma glucose levels of GK rats, whereas there are different effects on the expression of liver GLUT2 and GCK. In summary, the study indicates that SG and DJB are alternative methods of providing long-term control of glycemia and normal levels of insulin with better clinical advantages. In addition to providing good glycemic control, the results of both operations presented herein corroborate and extend previous work.

## Abbreviations

DJB, Duodenal-jejunal bypass; GCK, Glucokinase; GK, Goto–Kakizaki; OGTT, Oral glucose tolerance test; RT-PCR, Reverse transcription-PCR; SG, Sleeve gastrectomy.

## Competing interests

We declare that we have no competing interests.

## Authors’ contributions

ZD and LL participated in the design of this study, and they both performed the statistical analysis. JX carried out the study, together with ZC, and collected important background information, and drafted the manuscript. DW conceived of this study, and participated in the design and helped to draft the manuscript. All authors read and approved the final manuscript.
